# Cases Extracted from the Note-Book of Henry Davies, M.D.

**Published:** 1835-01-01

**Authors:** 


*Cases extracted from the Note-book of* HENRY DAVIES, M.D. Physician to the British Lying-in Hospital, &c.

### CASES OF RETAINED PLACENTA.

### I.

FEBRUARY 4, 1828. I was requested to see a young woman, some distance from town, who had been delivered of her first child early in the morning, after a natural but somewhat protracted labour. The placenta not following in a moderate space of time, repeated but fruitless attempts were made to extract it. The discharge was copious, but not so great as to be alarming. The patient being in considerable pain, an opiate was given her.

I saw her at two in the afternoon, eight hours after the birth of the infant. An enema had recently been given, and she was then tolerably tranquil, except that she was uneasy on account of the afterbirth (as she expressed it) not having come away; and her husband and friends were anxious and alarmed. The uterus was firm and contracted. I explained to them the nature of the case. Having taken the usual preliminary measures, and placed her *close to the edge* of the bed, and had a moderate pressure made by a bandage on the abdomen, I introduced my hand determinedly but cautiously into the uterus, and slowly detached the placenta from its upper and posterior part, and allowed the placenta to be expelled with my hand from the uterus. The operation occupied rather more than an hour from its commencement to its termination, the patient suffering a proportionate degree of pain.

She was now put to rights, and took a full dose of laudanum in a saline draught; which was repeated in moderate doses, and at distant intervals. She recovered without a bad symptom.

II.

September the 10th, 1833. I was requested by a midwife to see a patient, in the neighbourhood of Tottenham-court Road, who had been delivered between one and two o'clock in the morning, and in whom the placenta still remained undelivered, though repeated attempts to remove it had been made by the midwife and a neighbouring practitioner. As I was at the moment unable to attend, I wrote a note to a friend who resided near the patient, to request that he would see her in the mean time; and, when I got to the patient at one o'clock, I found that gentleman, with another, present, making great but unsuccessful exertions to pass his hand into the uterus, the patient expressing most agonizing pain. As I had been a witness of Mr. ——'s manual dexterity on former occasions, and as the external parts were very much swollen, and exquisitely tender, and the uterus firmly contracted, I did not deem it right to attempt to deliver the placenta; but it was agreed, in consultation, to give the woman, who was much exhausted and fatigued, a full dose of laudanum (ʒss. of Tr. Opii) in a saline draught, and follow it up with moderate doses through the night, to keep her tranquil, and to apply a large bread and water poultice to the pudenda. Early in the morning she was ordered an active aperient, (the compound senna draught,) which was to be repeated in four hours, if necessary; and, if this failed to act on the bowels, an enema was to be administered. By this treatment it was presumed that the patient would be composed, and that the uterus, having recruited its powers, would be induced to act sympathetically with the bowels.

There was one alvine evacuation before twelve o'clock, which was followed by some uterine pain, and about one o'clock the whole placental mass was expelled, with considerable pain, thirty-six hours from the time of the delivery of the child: it was entire and very large.

The pudenda were still poulticed, and it was requisite to draw off the urine for several days: the discharge copious and fœtid. On the third day she had a smart febrile attack, preceded by some rigor, and accompanied by an accelerated pulse, and abdominal pain. A dozen leeches were applied to the abdomen; three grains of calomel, with one of opium, were given every fourth hour; the Liq. Ammoniæ Acetatis was also administered; and the bowels were opened with Ol. Ricini.

These symptoms subsided on the fifth day after delivery, and the second after the attack, when a superficial abscess of some extent formed in the left hypochondriac region. This abscess did not appear directly connected with her parturient state, and she imagined that she had hurt her side against the side of the bed, in her struggles during labour. The abscess was opened, and its contents discharged.

From this time she very gradually recovered, but was not able to retain her urine for some weeks, nor to go out till the third month.

III.

The following case I saw in consultation. The patient, a lady, aged thirty-two, was delivered of her first child on the 17th of May, 1834. The practitioner who originally attended says, "the pains became so weak and ineffective, that, after remaining eight hours, I gave Secalis cornuti ℈ss. in a cup of tea: within an hour afterwards the child was born. The placenta was attached so firmly, that, after the lapse of an hour and ten minutes, I passed my hand into the uterus, and so completed with ease the separation. During the time of my attendance, there was but little discharge of any kind."

A piece of placenta was expelled from the vagina on the 19th; the lochia were first observed to be fœtid on the 20th; and from this time till the death of the patient, the pulse varied from 90 to 130.

It would be needless to give all the symptoms detailed in Mr. ——'s account of the case, and still more fruitless to copy the prescriptions; let it suffice to say, that the patient died on the 6th of June, exhausted by irritative fever, and that the treatment consisted chiefly of the exhibition of opium, ammonia, and aromatics, with effervescing draughts, and occasional aperients.

This patient was visited once by a physician of great eminence.

*Post-mortem Examination,* June 8th, Six A.M. On laying open the abdominal parietes, the viscera and peritoneal covering of the uterus appeared free from disease; the uterus had sunk into the pelvis, and was of the usual size of that organ three weeks after delivery. The bladder contained half a pint of pale urine. The uterus, with its appendages, the bladder, and the greater portion of the vagina, being removed, the whole externally appeared healthy. The ovaria were sound, and there was a well-marked corpus luteum. On cutting open the uterus by a longitudinal incision throughout its whole length, a portion of the placenta was seen firmly but not morbidly adherent to the upper and posterior part of the fundus, with a portion hanging down below the os uteri. The portion attached to, and that within the cavity of the uterus, was healthy in its structure; but the part in contact with and external to the os uteri was putrid, and the substance of the os uteri was of a peculiar bluish black colour, soft, and easily torn. The veins in the substance of the uterus contained pus and black coagulated blood.

At the upper part of the right hepatic region was an abscess, communicating with the (right) thoracic cavity, which was full of sero-purulent fluid, pressing the lung laterally against the mediastinum. The surface of the lung was covered with a thick layer of coagulable lymph ; it was condensed in its substance, and at its inferior part were several abscesses containing pus. A portion of the inferior lobe and pulmonary texture was destroyed by gangrene, the structure surrounding which was of a deep red colour, dense, and infiltrated with pus.

The practical conclusions to be drawn from these cases are, that the placenta, when not expelled by the natural powers, aided by the usual assistance, should be delivered by the hand passed into the uterus, after having waited such a space of time (say two hours,) that the woman's powers are recruited, and the uterus has recovered its tone, yet before it is firmly contracted. If this fails to effect the delivery, after every due and proper exertion has been made, it is better to leave the whole than a part of the placenta, as the uterus is more likely to be excited to act on the larger than the smaller mass.

We also see that, in the event of untoward symptoms arising, it is always better to make an examination *per vaginam,* and if any portion of the placenta be detected, or if there be a probability of any portion being in utero, in addition to attending to the general symptoms, some astringent and sedative injection should be thrown up, at short intervals, such as solutions of alum, or of any of the mineral acids. This serves both to wash away the putrid matter, the absorption of which is thought to give rise to the train of fatal symptoms; and, by keeping as it were the lumps of placenta macerated, such injections possibly may produce the contraction of their volume, and accelerate their detachment from the uterine surface.

On the subject of retained placenta much useful information may be gained by the perusal of Dr. Douglas's pamphlet on the Hourglass Contraction of the Uterus, Dr. Murdoch on Retained Placenta, and the first part of Dr. Ramsbotham's Observations on Midwifery.

